# The Treatment of the Puerpeirum

**Published:** 1913-06-28

**Authors:** 


### MATERNITY AND MOTHERING.

(Inquiries and Correspondence are invited.)

### The Treatment of the Puerperium.

There is, it may be supposed, an innate tendency of physicians, which they have to be constantly repressing, to pay less attention to the supervision of their puerperal patients than to that of the actual processes of labour itself. We think this comparative lack of interest is less pronounced in this country than it is elsewhere; but even here it is known to exist in the practice of a small percentage of obstetricians. In the vast majority of cases a capable nurse or midwife safeguards the patient from any harm, both by herself doing that which is needful and by informing the practitioner promptly of any departure from the normal. But it has to be borne in mind that an enormous number of confinements never come into the hands of a medical practitioner at all, and that of necessity the whole-time services of a duly qualified midwife or skilled maternity nurse are not available for great numbers of mothers of the labouring class. What happens is that, in consequence of this, many of the bad old customs handed down by incompetent Gamps are perpetuated and practised still, in spite of the example and precept dinned into their ears by the medical profession for many years.

It has been stated that medical men themselves fail sometimes to watch over the puerperium with that eagle eye which experience shows to be almost as important as skilled management of the actual details and conduct of labour itself. As far as the medical profession is concerned the remedy lies in the hands of obstetric teachers and examiners. Not many errors of commission can be laid to the charge of the profession as a whole: more often it is the omission to give specific directions to a not over-competent nurse that results in puerperal abnormalities. To attempt here to compile a list of directions for the woman—be she midwife, nurse, or neighbour—in charge of a puerperal patient to follow would be too large a task; a text-book would be necessary to contain them all. But there are certain points, many of them individually of small moment, which experience has shown the writer are not infrequently forgotten or misunderstood. Duly trained midwives or nurses will hardly require to be reminded of any of these fairly elementary rules—but then, as we have already pointed out, vast numbers of the nation's mothers cannot command these skilled services.

To begin with, let us assume that a confinement is safely over, without any unusual difficulty. Not so very long ago there was a custom of depriving the mother of her pillow for twenty-four hours, and of making her wait another like period before allowing both bolster and pillow. This custom is rightly falling into disuse. It is not usual in this country to follow the custom of some German obstetricians of allowing new-made mothers to sit up or get up within a day or two of confinement; but to go to the other extreme is often to inflict a quite unnecessary hardship. As for position there is much to be gained by abandoning the old plan of insisting on the patient lying flat on her back. Indeed she should spend at least half her time on one side or the other; and her posture should be changed fairly frequently, except of course during sleep.

Then a daily wash all over is essential: not necessarily all at one time during the first three or four days, but piecemeal if the patient is very tired or exhausted. The actual external genitals should be swabbed over gently but thoroughly with clean swabs soaked in lysol solution (a teaspoonful to the pint of water); care to be taken to wipe always backwards, and not to use any swab again which has once touched the region about the anus. Whenever the bowels have acted, or urine has been passed, it is a good plan to pour some boiled and cooled water over the parts. Before embarking on any of these proceedings, the attendant should wash her own hands thoroughly with soap and water, and go straight to the patient without drying the hands.

Care should be taken that the mother is able to urinate three times at least in the twenty-four hours. As a rule the only difficulty in securing this arises on the first, or first and second, days of the lying-in. Hot fomentations placed on the lower part of the abdomen sometimes help; and another dodge which answers sometimes is pouring water from one vessel into another, so that the sound of trickling fluid can be heard by the patient. If neither of these plans answers, the attendant should leave the room for two or three minutes, as sometimes her presence seems to inhibit the function of a shy patient. Failing this, the patient may be allowed to kneel in bed, carefully supported, for a short time. If by none of these means urination can be accomplished, the doctor will have to be acquainted with the situation. On no account is any vaginal douche to be given or any vaginal examination made without the express consent and directions of the doctor.

As for diet, here again modern practice has broken quite away from the regimen which was venerated a generation or two back. Instead of keeping the mother strictly on gruel and such like meagre fare for some days, she is now allowed a much more satisfying and nutritious diet. Some doctors still object to solid food until the bowels have acted, usually on the third day; but many now allow fish and even fowl before that, say on the second or third day. With regard to visitors, restrictions should be stoutly maintained. For twenty-four hours after a confinement no one should be allowed in except the patient's husband, and he only for five minutes at a time. After that, for the next four or five days, the quieter the patient will consent to keep the better; and at the outside one visitor a day should be allowed as a rule. Some women, no doubt, will rebel against this and fret themselves if some relaxation of the rules be not allowed; but most patients seem rather glad than otherwise to have visitors kept firmly at a distance. These are a few of the more important principles to keep in mind, as far as the mother is concerned.

